# *EXO70B1* Modulates Dark-Induced Leaf Senescence in an Age-Dependent Manner Associated with *NYE1*-Dependent Chlorophyll Catabolism

**DOI:** 10.3390/plants15101461

**Published:** 2026-05-11

**Authors:** Junkui Xie, Siyu Ji, Tianyu Zhu, Yanli Cheng, Yaqi Wang, Guangyou Duan, Shan Gao

**Affiliations:** 1 Key Laboratory of Biological Resources and Ecology of Pamirs Plateau in Xinjiang Uygur Autonomous Region, College of Life and Geographic Sciences, Kashi University, Kashi 844000, China; xiejunkui0429@outlook.com; 2 School of Life Sciences, Qilu Normal University, Jinan 250200, China; m15054011635@163.com (S.J.); 19157101725@163.com (T.Z.); 13173290913@163.com (Y.C.); 18906381757@163.com (Y.W.); guangyou.duan@qlnu.edu.cn (G.D.)

**Keywords:** dark-induced leaf senescence, *EXO70B1*, age-dependent, *NYE1*, *Arabidopsis thaliana*

## Abstract

Dark-induced senescence (DIS) is a coordinated physiological process associated with chlorophyll degradation, macromolecular turnover, and nutrient remobilization under prolonged darkness. *EXO70B1*, a subunit of the exocyst complex, has been implicated in intracellular membrane trafficking, autophagy-associated vacuolar transport, and salicylic acid-dependent immunity. However, whether *EXO70B1* contributes to DIS remains unknown. Here, we show that *EXO70B1* expression increases with leaf age and is transiently induced during the early phase of dark treatment. Accordingly, as a consequence of loss of *EXO70B1*, an acceleration of dark-induced leaf yellowing, chlorophyll degradation, and decline in photosynthetic performance was observed. Notably, this hypersensitivity was strongly age-dependent, being evident in mature (4-week-old) plants but not in younger plants. Genetic analyses indicated that the accelerated chlorophyll degradation in the *EXO70B1* mutant background depends on *NYE1* function. To investigate the molecular basis underlying this age-specific transition, we performed stage-resolved transcriptomic profiling, which identified the 4-week stage as a major point of divergence between Col-0 and *exo70b1-1*. Before visible necrosis, mature *exo70b1-1* leaves displayed substantial transcriptional reprogramming, including enrichment of salicylic acid (SA) signaling, systemic acquired resistance (SAR), and other defense-related pathways. Collectively, our findings support a role for *EXO70B1* as an age-dependent modulator of DIS and indicate that the enhanced dark sensitivity of mature *exo70b1-1* leaves is associated with defense-related transcriptional reprogramming and *NYE1*-dependent chlorophyll degradation.

## 1. Introduction

Leaf senescence is the terminal phase of leaf development and a genetically programmed, actively regulated transition that is essential for nutrient recycling and plant fitness [1,2,3,4]. This process is governed by the integration of endogenous developmental programs and environmental cues. Internal factors, including phytohormone status and developmental age, act together with external inputs such as nutrient availability, light regime, and abiotic stresses (e.g., heat, drought, and salinity) to fine-tune the onset and progression of senescence [5,6]. Biotic signals, particularly pathogen infection, further influence its timing and pace [7,8,9]. Accordingly, leaf senescence is broadly categorized into age-dependent senescence and stress-induced senescence, the latter serving as an adaptive strategy to optimize resource reallocation under adverse conditions [10,11].

At the physiological level, senescence is characterized by a decline in photosynthetic capacity, extensive chlorophyll degradation, and progressive chloroplast dismantling [12,13,14]. In parallel, proteins, lipids, and nucleic acids are systematically catabolized to mobilize nutrients from senescing tissues to developing sink organs [4,15,16]. An important contributor to this metabolic transition is macroautophagy (hereafter referred to as autophagy), a conserved eukaryotic degradation pathway in which cytoplasmic materials are sequestered into double-membrane autophagosomes and delivered to the vacuole for recycling [17,18,19,20]. The importance of autophagy in nutrient remobilization is underscored by autophagy-defective mutants, such as *atg2*, *atg5*, and *atg7*, which exhibit hypersensitivity to carbon starvation, premature chlorosis, and reduced growth duration [19,21,22]. Although substantial progress has been made in defining the core machinery of autophagosome biogenesis [23,24], how senescence-associated recycling interfaces with membrane tethering and vesicle trafficking systems remains incompletely understood.

Efficient intracellular vesicle trafficking is required for vacuolar delivery and recycling of senescence-associated cargoes, including processes linked to autophagy [14,23,25]. One major tethering machinery is the exocyst, an evolutionarily conserved octameric complex composed of SEC3, SEC5, SEC6, SEC8, SEC10, SEC15, EXO70, and EXO84, which classically mediates the targeting of secretory vesicles to specific membrane domains prior to membrane fusion [26]. Unlike yeast and animals, the plant EXO70 subunit family has undergone extensive expansion, driving highly diversified exocyst functions [27,28]. In *Arabidopsis thaliana*, this expansion has produced 23 EXO70 paralogs with highly tissue- and cell-type-specific expression patterns [29]. The distinct subcellular microdomain localization of these isoforms suggests substantial functional specialization [30,31], enabling precise coordination of specialized endomembrane trafficking routes required for polar secretion, development, and stress adaptation [28,32]. Together, these observations raise the possibility that different EXO70 isoforms define specialized exocyst subcomplexes or trafficking pathways in plants [29,33].

Among these, *EXO70B1* has emerged as a functionally important member of the *Arabidopsis thaliana EXO70* family. Previous studies showed that EXO70B1 interacts with core exocyst subunits such as SEC5 and EXO84 and has been linked to ATG8-associated trafficking processes and vacuolar transport. Consistent with this, *exo70b1* mutants display nutrient- and energy-dependent spontaneous leaf lesions, reminiscent of phenotypes observed in autophagy mutants, as well as defects in vacuolar import of secondary metabolites [24,25,34,35,36]. In addition, *EXO70B1* has been associated with salicylic acid (SA)-related defense signaling and autoimmune-like phenotypes, suggesting broader roles in maintaining cellular homeostasis [34,37,38]. Genetic analyses further showed that the spontaneous cell-death phenotype of *exo70b1* can be suppressed by mutations in immune regulators such as *TN2* and *NPR1*, indicating that *EXO70B1* is connected to pathways that restrain inappropriate defense activation [24,34,36]. Moreover, *EXO70B1* has been implicated in trafficking events related to plasma membrane immune components, including the pattern-recognition receptor FLS2 [38]. However, although previous work has linked *EXO70B1* to *SA*-dependent immunity and defense regulation [33,34,37], a role in leaf senescence has not been established.

Darkness provides a robust and widely used experimental system for studying leaf senescence, as it suppresses photosynthetic carbon fixation and promotes chlorophyll degradation, chloroplast dismantling, and nutrient remobilization [39,40,41]. Chlorophyll catabolism is a hallmark of this process, and *NON-YELLOWING1/STAY-GREEN1* (*NYE1/SGR1*) encodes a Mg-dechelatase that catalyzes the removal of Mg^2+^ from chlorophyll a to produce pheophytin a [42,43]. *NYE1* also facilitates the recruitment or assembly of other chlorophyll catabolic enzymes, including NYC1, NOL, and PAO, at light-harvesting complex II (LHCII), thereby promoting chlorophyll–protein complex destabilization during chlorophyll breakdown [13,44,45]. However, whether membrane trafficking pathways contribute to chlorophyll degradation during DIS remains unclear. In this study, we investigated whether the exocyst subunit *EXO70B1* modulates DIS in *Arabidopsis thaliana*. We show that loss of *EXO70B1* accelerates DIS in an age-dependent manner. By analyzing the *exo70b1-1* mutant and constructing the *exo70b1-1 nye1-1* double mutant, we examined the genetic relationship between *EXO70B1* and *NYE1* in dark-induced chlorophyll degradation. Furthermore, using age-resolved transcriptomic analysis, we sought to characterize the transcriptional reprogramming events preceding visible damage and to assess whether the accelerated DIS phenotype in *exo70b1* is associated with defense-related transcriptional changes.

## 2. Results

### 2.1. EXO70B1 Transcript Abundance Increases During Leaf Aging

**Figure 1:**
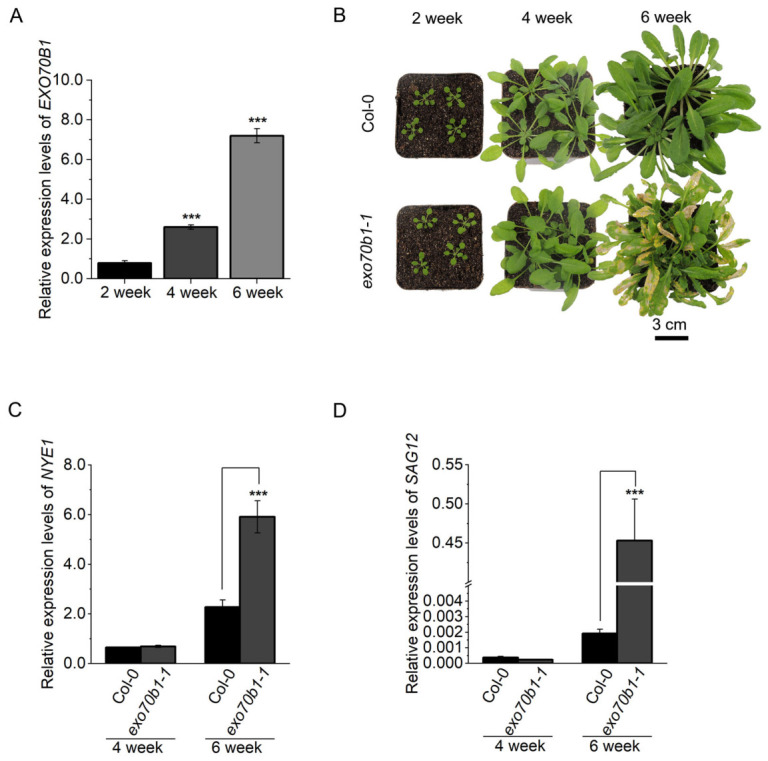
*EXO70B1* expression rises with leaf age. (**A**) Expression analysis of the *EXO70B1* gene in leaves of Col-0 plants at different ages (2, 4, and 6 weeks) under short-day conditions. Expression levels were determined by RT-qPCR using the 5th and 6th rosette leaves. (**B**) Overall phenotypes of 2-, 4-, and 6-week-old Col-0, *exo70b1-1* mutant plants grown under short-day conditions. Scale bar = 3 cm. (**C**,**D**) Expression analysis of the key chlorophyll catabolic regulator *NYE1* (**C**) and the senescence marker gene *SAG12* (**D**) in leaves of Col-0 and *exo70b1-1* mutants at different ages (4 and 6 weeks) under short-day conditions. The 5th and 6th rosette leaves were used for analysis. Transcript levels were determined by RT-qPCR using *ACTIN2* as an internal control. Data are presented as mean ± SD (*n* = 3 biological replicates). Statistical significance was determined by one-way ANOVA followed by Tukey’s multiple comparison test; asterisks indicate significant differences, *** *p* < 0.001).

Previous studies have implicated the exocyst subunit *EXO70B1* in plant immunity and nutrient-stress responses. In addition, *exo70b1* mutants display starvation-dependent spontaneous lesions and SA overaccumulation, suggesting broader alterations in cellular homeostasis [24]. We therefore asked whether *EXO70B1* is associated with leaf senescence. Publicly available transcriptomic data from the *Arabidopsis thaliana* eFP Browser: https://www.bar.utoronto.ca/ (accessed on 1 August 2025) [46] showed that *EXO70B1* expression was elevated in senescing leaves (Figure S1). Consistent with this trend, reverse transcription quantitative polymerase chain reaction (RT-qPCR) analysis of the 5th and 6th rosette leaves showed that *EXO70B1* transcript levels were higher at 4 weeks than at 2 weeks, and further increased at 6 weeks (Figure 1A), indicating an age-associated rise in *EXO70B1* expression.

To test the physiological relevance of *EXO70B1*, we compared wild type (Col-0) and the null mutant *exo70b1-1* (GK-114C03) under short-day conditions. No obvious phenotypic differences were observed at 2 and 4 weeks. However, by 6 weeks of age, the *exo70b1-1* mutant exhibited premature yellowing together with spontaneous necrotic lesions on mature leaves, consistent with previous reports [34] (Figure 1B). At the molecular level, transcript levels of the chlorophyll catabolic regulator *NYE1* and the senescence-associated marker gene *SAG12* were significantly higher in 6-week-old *exo70b1-1* leaves than in age-matched Col-0 plants (Figure 1C,D). Together, these data show that *EXO70B1* expression increases with leaf age, and the *exo70b1* mutant exhibits premature age-associated leaf yellowing accompanied by increased expression of senescence-associated marker genes.

### 2.2. Loss of EXO70B1 Accelerates Dark-Induced Leaf Senescence

**Figure 2:**
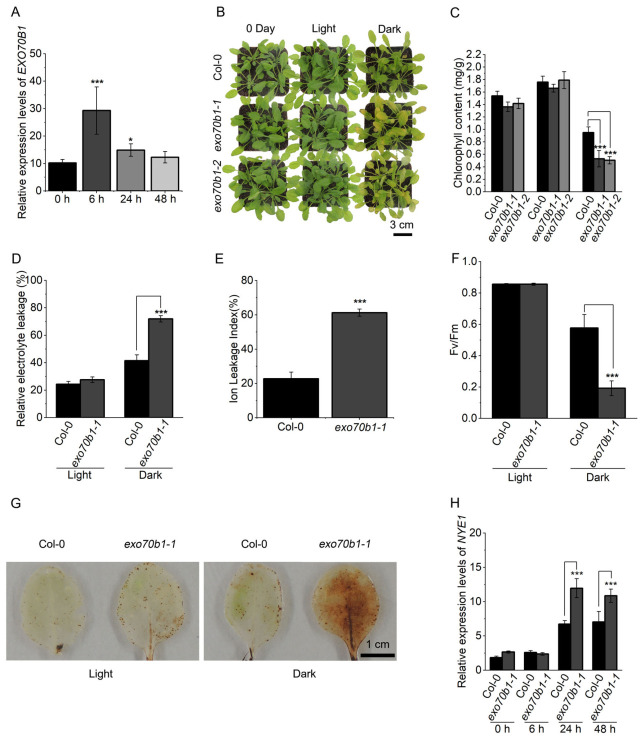
Loss of *EXO70B1* accelerates DIS. (**A**) Time-course analysis of *EXO70B1* expression in Col-0 leaves under dark treatment. Samples were collected at 0, 6, 24, and 48 h after transfer to darkness. Transcript levels were determined by RT-qPCR using *ACTIN2* as an internal control. (**B**) Phenotypes of 4-week-old Col-0, *exo70b1-1* and *exo70b1-2* mutant plants at 0 days and after 5 days of light or dark treatment. Scale bar = 3 cm. (**C**) Chlorophyll content corresponding to (**B**). (**D**,**E**) Relative electrolyte leakage after 5 days of light or dark treatment (**D**) and the dark-induced absolute ion leakage index (**E**) in the 5th and 6th rosette leaves of Col-0 and *exo70b1-1*. (**F**) *Fv*/*Fm* in the 5th and 6th rosette leaves of Col-0 and *exo70b1-1* after 5 days of light or dark treatment. (**G**) DAB staining showing H_2_O_2_ accumulation in the 5th and 6th rosette leaves of Col-0 and *exo70b1-1* after 3 days of light or dark treatment. (**H**) *NYE1* expression in the 5th and 6th rosette leaves of Col-0 and *exo70b1-1* at 0, 6, 24, and 48 h during dark treatment. Transcript levels were determined by RT-qPCR using *ACTIN2* as an internal control. Data are presented as mean ± SD (*n* = 5 biological replicates for chlorophyll measurements, *n* = 8 for *Fv*/*Fm*, and *n* = 3 for all other assays). Statistical significance was determined by one-way ANOVA followed by Tukey’s multiple comparison test (* *p* < 0.05, *** *p* < 0.001).

Dark treatment is widely used to trigger rapid and synchronized senescence by imposing acute carbon deprivation. To test whether *EXO70B1* participates in this process, we first examined its transcriptional response to darkness in the 5th and 6th rosette leaves of 4-week-old Col-0 plants. RT-qPCR showed that *EXO70B1* transcripts increased markedly at 6 h after dark transfer, then declined by 24 h and returned to near basal levels by 48 h (Figure 2A). This transient induction indicates that *EXO70B1* responds rapidly to dark treatment and suggests possible involvement in early events during DIS.

We next compared DIS progression in Col-0 and two independent loss-of-function alleles, *exo70b1-1* (GK-114C03) and *exo70b1-2* (GK-156G02). After 5 days under identical dark conditions, both mutants displayed more severe yellowing than Col-0, accompanied by a greater reduction in total chlorophyll content (Figure 2B,C). A detached-leaf dark assay yielded similar results, with more rapid yellowing and chlorophyll loss in *exo70b1* mutants than in Col-0 (Figure S2A,B).

To evaluate the extent of tissue deterioration, we measured membrane integrity and photosynthetic performance. After 5 days of dark treatment, *exo70b1-1* exhibited significantly higher relative and absolute ion leakage than Col-0, indicating greater membrane damage (Figure 2D,E). Concurrently, the maximal quantum efficiency of PSII (*Fv*/*Fm*) declined more strongly in *exo70b1-1* than in Col-0 (Figure 2F). After 3 days of dark treatment, 3,3′-Diaminobenzidine (DAB) staining revealed higher H_2_O_2_ accumulation in *exo70b1-1* leaves than in Col-0 (Figure 2G), suggesting increased oxidative stress in the mutant under darkness.

At the molecular level, *NYE1* expression was significantly higher in *exo70b1-1* than in Col-0 at both 24 h and 48 h of dark treatment (Figure 2H). Together, these phenotypic, physiological, and transcriptional data show that loss of *EXO70B1* is associated with accelerated dark-induced leaf yellowing, increased cellular damage, and enhanced senescence-associated gene expression.

### 2.3. The Accelerated Dark-Induced Senescence Phenotype of exo70b1 Is Age Dependent

Previous studies showed that *exo70b1* plants develop spontaneous necrotic lesions only after 4 weeks of growth [24,34], suggesting that some *exo70b1*-associated phenotypes are developmentally dependent. We therefore investigated whether the effect of *EXO70B1* loss on DIS is also age-dependent.

**Figure 3:**
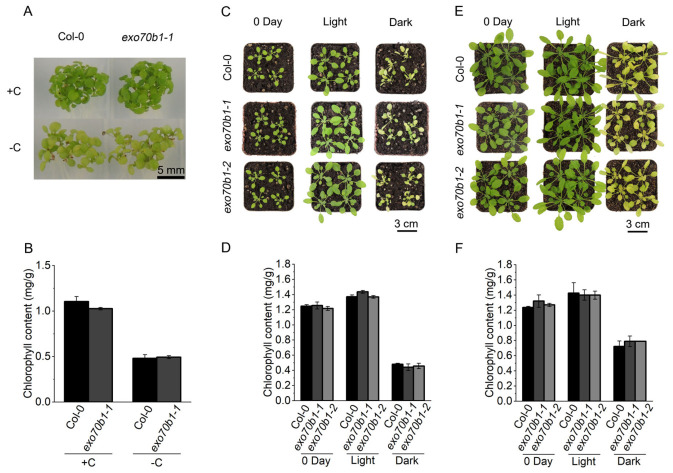
Age-dependent effects of *EXO70B1* loss on DIS-associated phenotypes. (**A**) Phenotypes of 7-day-old Col-0 and *exo70b1-1* seedlings after 5 days of dark treatment on sugar-free medium (−C). Seedlings grown under light on sucrose-containing medium (+C) were used as controls. (**B**) Chlorophyll content of seedlings shown in (**A**). (**C**) Phenotypes of 2-week-old Col-0, *exo70b1-1* and *exo70b1-2* plants at 0 days and after 5 days of light or dark treatment. Scale bar = 3 cm. (**D**) Chlorophyll content corresponding to (**C**). (**E**) Phenotypes of 3-week-old Col-0, *exo70b1-1* and *exo70b1-2* plants at 0 days and after 5 days of light or dark treatment. Scale bar = 3 cm. (**F**) Chlorophyll content corresponding to (**E**). Data are presented as mean ± SD (*n* = 5 biological replicates). Statistical significance was determined by one-way ANOVA followed by Tukey’s multiple comparison test.

When 7-day-old seedlings were transferred to sugar-free medium and incubated in darkness for 5 days, chlorophyll content declined rapidly and to a similar extent in Col-0 and *exo70b1-1* (Figure 3A,B). Similarly, dark treatment of 2- and 3-week-old soil-grown plants for 5 days yielded no significant differences between Col-0 and *exo70b1* mutants in visible yellowing or chlorophyll content (Figure 3C–F). In contrast, more pronounced yellowing and greater chlorophyll loss were evident in 4-week-old *exo70b1* plants (Figure 2). These findings indicate that the accelerated DIS in *exo70b1* mutants is not a general property of the genotype, but depends on developmental stage. Thus, the effect of *EXO70B1* loss on DIS becomes evident only at later developmental stages.

### 2.4. Genetic Complementation with EXO70B1 Rescues the Accelerated Dark-Induced Phenotype of exo70b1-1

**Figure 4:**
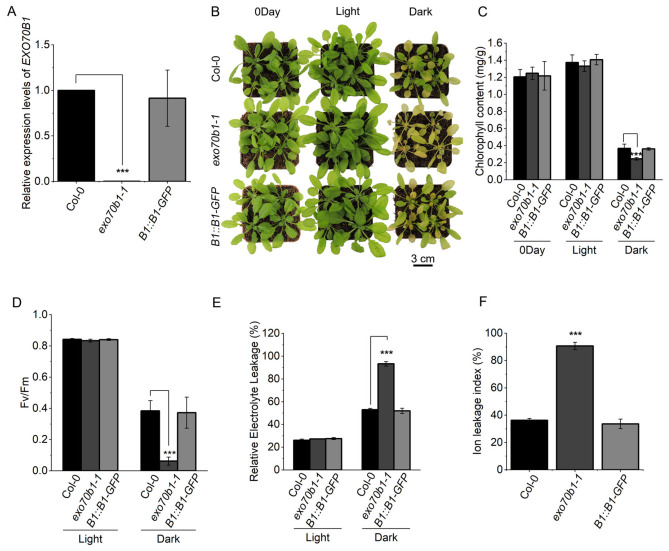
Genetic complementation with *EXO70B1* restores the dark-induced phenotype of *exo70b1-1*. (**A**) *EXO70B1* expression in Col-0, *exo70b1-1* and complementation line *B1::B1-GFP*. Transcript levels were determined by RT-qPCR, normalized to *ACTIN2*, and expressed relative to Col-0 using the 2^−ΔΔCt^ method. (**B**) Phenotypes of 4-week-old Col-0, *exo70b1-1* and *B1::B1-GFP* plants at 0 days and after 5 days of light or dark treatment. (**C**–**F**) Quantification of physiological parameters in the 5th and 6th rosette leaves shown in (**B**), including total chlorophyll content (**C**), the maximum photochemical efficiency of PSII (Fv/Fm) after 5 days of light or dark treatment (**D**), relative electrolyte leakage after 5 days of light or dark treatment (**E**), and the dark-induced absolute ion leakage index (**F**). Data are presented as mean ± SD (*n* = 3 biological replicates for RT-qPCR and ion leakage measurements, *n* = 5 for chlorophyll measurements, and *n* = 6 for *Fv*/*Fm*). Statistical significance was determined by one-way ANOVA followed by Tukey’s multiple comparison test. Asterisks indicate significant differences (*** *p* < 0.001).

To verify that the accelerated dark-induced phenotype is caused by loss of *EXO70B1*, we generated a complementation line expressing *pEXO70B1::EXO70B1*-GFP in the *exo70b1-1* background (hereafter *B1::B1-GFP*). RT-qPCR showed that *EXO70B1* transcript abundance in the complementation line was restored to near Col-0 levels (Figure 4A). Under short-day conditions, spontaneous lesions seen in *exo70b1-1* were abolished in the complementation line (Figure S3), indicating functional rescue.

After 5 days of dark treatment, the *B1::B1-GFP* line displayed leaf yellowing comparable to that of Col-0, and the accelerated yellowing phenotype observed in *exo70b1-1* was no longer evident (Figure 4B). Quantitative measurements further supported this rescue: total chlorophyll, *Fv*/*Fm*, and ion leakage in the complementation line were similar to those in Col-0 (Figure 4C–F). Together, these results provide genetic evidence that the accelerated dark-induced phenotype of *exo70b1-1* is attributable to loss of *EXO70B1*.

### 2.5. NYE1 Is Required for Accelerated DIS in exo70b1 Mutant

**Figure 5:**
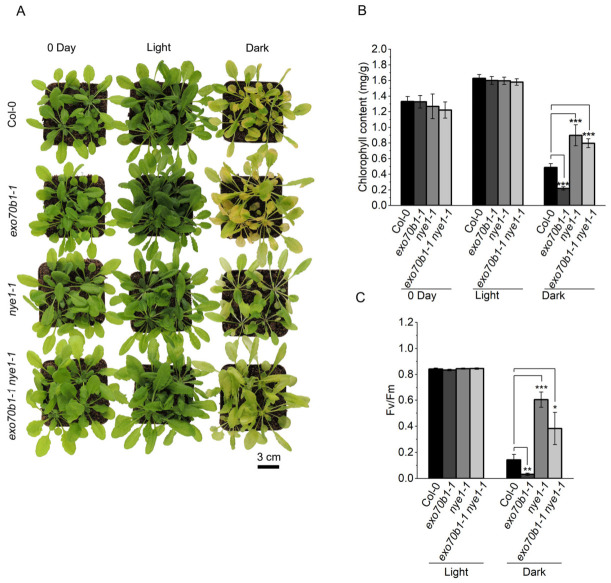
Loss of *NYE1* suppresses accelerated DIS in *exo70b1-1*. (**A**) Phenotypes of 4-week-old Col-0, *exo70b1-1*, *nye1-1* and *exo70b1-1 nye1-1* plants at 0 days and after 5 days of light or dark treatment. Scale bar = 3 cm. (**B**) Total chlorophyll content corresponding to (**A**). (**C**) *Fv*/*Fm* in the 5th and 6th rosette leaves measured after 5 days of light or dark treatment, corresponding to panel (**A**). Data are presented as mean ± SD (*n* = 5 biological replicates). Statistical significance was determined by one-way ANOVA followed by Tukey’s multiple comparison test. Asterisks indicate significant differences (* *p* < 0.05, ** *p* < 0.01, *** *p* < 0.001).

To investigate the genetic relationship between *EXO70B1* and the chlorophyll degradation regulator *NYE1*, we generated the *exo70b1-1 nye1-1* double mutant. After 5 days of dark treatment, the double mutant displayed a pronounced stay-green phenotype compared with Col-0 and *exo70b1-1*, and was visually similar to *nye1-1* (Figure 5A). Consistent with this phenotype, chlorophyll content and *Fv*/*Fm* were substantially higher in the double mutant than in Col-0 or *exo70b1-1* (Figure 5B,C). Notably, no significant difference in chlorophyll content was detected between *nye1-1* and *exo70b1-1 nye1-1* under these conditions (Figure 5B). These genetic data indicate that the accelerated chlorophyll loss phenotype of *exo70b1-1* during dark treatment is largely dependent on *NYE1*.

Under short-day conditions without dark treatment, the double mutant still developed spontaneous lesions, although lesion severity was reduced relative to *exo70b1-1* (Figure S4). This result suggests that, under normal growth conditions, additional processes independent of *NYE1* contribute to lesion formation, and that not all *exo70b1*-associated phenotypes can be explained by the *NYE1*-dependent chlorophyll degradation pathway.

### 2.6. Stage-Specific Transcriptomic Changes and Stress Pathway Activation in exo70b1-1

To identify molecular events associated with the enhanced DIS phenotype in *exo70b1-1*, we performed RNA sequencing (RNA-seq) on rosette leaves from 3- and 4-week-old Col-0 and *exo70b1-1* plants grown under short-day conditions. Notably, at 4 weeks, *exo70b1-1* remains phenotypically indistinguishable from Col-0 under standard short-day growth conditions, yet already shows enhanced sensitivity to dark treatment, prompting us to ask whether transcriptomic differences emerge during this developmental window.

**Figure 6:**
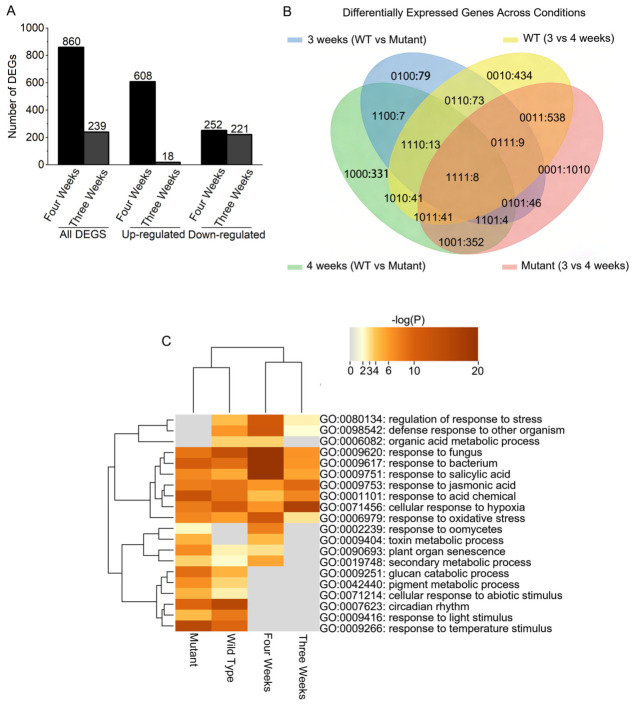
Age-specific differential gene expression and GO enrichment analysis in *exo70b1-1* mutant. (**A**) Bar chart quantifying total, up-, and down-regulated DEGs in 3-week and 4-week WT vs. mutant comparisons. (**B**) Venn diagram showing DEGs (|log_2_FC| ≥ 1.5, padj ≤ 0.01) among four comparisons: 3-week WT vs. mutant, 4-week WT vs. mutant, WT developmental (3w vs. 4w), and mutant developmental (3w vs. 4w). DEG analysis used nf-core framework (rnaseq v3.22.2, differentialabundance v1.5.0) with Ensembl Plants release 62 annotation. Complete results are in Table S2. (**C**) Heatmap of GO biological process enrichment for DEGs across the four comparisons (3-week WT vs. mutant, 4-week WT vs. mutant, WT developmental, mutant developmental). Color intensity represents −log_10_(*p* value). GO enrichment was performed with Metascape v3.5.20260201.

Differential expression analysis (WT vs. mutant) revealed a marked age-dependent expansion of transcriptional divergence (Figure 6A). At 3 weeks, 239 DEGs were detected, whereas at 4 weeks this number increased to 860. This >3-fold increase is consistent with substantially greater transcriptomic divergence at 4 weeks.

Multi-condition Venn analysis further highlighted stage divergence (Figure 6B). Most DEGs were stage-specific, with 828 genes unique to the 4-week comparison and 1412 genes changing exclusively during the 3-to-4-week transition in the *exo70b1-1* background. Together, these patterns are consistent with age-dependent transcriptional changes associated with loss of *EXO70B1*.

To gain insight into the biological processes associated with these transcriptional changes, we performed Gene Ontology (GO) enrichment analyses (Figure 6C). DEGs associated with the 4-week mutant state were significantly enriched for “regulation of response to stress,” “response to salicylic acid,” and “response to jasmonic acid.” Notably, “plant organ senescence” was specifically enriched during the 4-week mutant transition, consistent with the emergence of the age-dependent dark-sensitive phenotype. By contrast, 3-week samples showed more limited enrichment of these stress-related categories. Terms related to “circadian rhythm” and “response to light stimulus” were enriched in both genotypes, although their enrichment patterns differed between Col-0 and *exo70b1-1*. Collectively, these data show that the *exo70b1-1* transcriptome diverges more strongly from that of Col-0 at 4 weeks than at 3 weeks, with enrichment of stress-, hormone-, and senescence-related progresses. These transcriptomic changes are associated with the developmental stage at which the dark-sensitive phenotype becomes apparent. Whether they contribute directly to this phenotype or instead reflect downstream consequences of *EXO70B1* loss remains to be determined.

### 2.7. Defense- and SA/SAR-Related Pathways Are Enriched in 4-Week-Old exo70b1-1 Prior to Visible Necrosis

At 3 weeks, most DEGs were downregulated in the mutant (221/239, 92.5%; WT vs. mutant). By 4 weeks, this pattern reversed: 608 genes were upregulated and 252 were downregulated in *exo70b1-1* (Figure 6A), indicating a marked shift in transcriptomic state.

**Figure 7:**
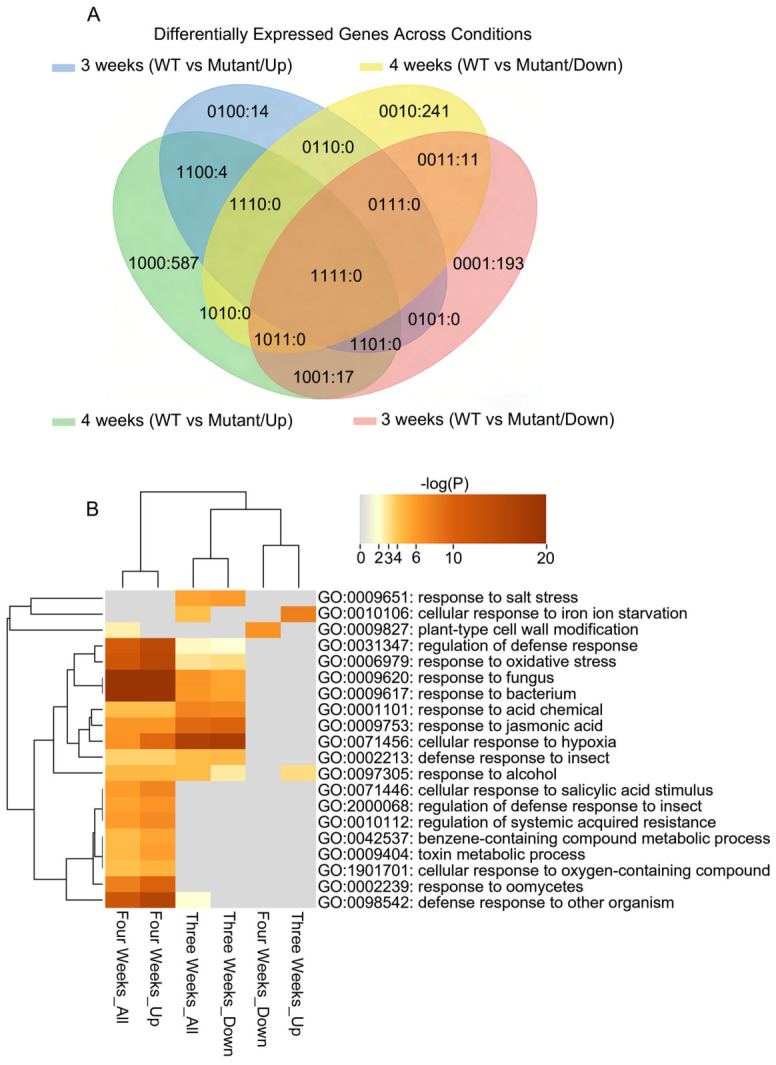
Stage-Specific Defense Pathway Enrichment in *exo70b1-1* Mutants Revealed by Transcriptomic Analysis. (**A**) Venn diagram showing overlap of up- and down-regulated DEGs between 3-week and 4-week WT vs. mutant comparisons. Numbers indicate unique or shared DEGs. DEG analysis used nf-core framework (rnaseq v3.22.2, differentialabundance v1.5.0) with Ensembl Plants release 62 annotation. Complete results are in Table S2. (**B**) Heatmap of GO biological process enrichment for up- and down-regulated DEGs separately in 3-week and 4-week WT vs. mutant comparisons. Color intensity represents −log_10_(*p* value). GO enrichment was performed with Metascape v3.5.20260201.

To further resolve stage-specific signatures, we performed multi-set Venn analysis (Figure 7A). Overlap between 3-week and 4-week DEG sets was minimal, indicating that the 4-week *exo70b1-1* transcriptome differs substantially from that at 3 weeks. Notably, 604 genes were uniquely upregulated in the mutant at 4 weeks, whereas 193 genes specifically downregulated at 3 weeks did not remain differentially expressed at 4 weeks.

GO enrichment analysis of 4-week upregulated gene set (Figure 7B) showed prominent enriched for defense-related processes. The 4-week upregulated gene set (Four Weeks_Up) was highly enriched for “regulation of systemic acquired resistance (SAR),” “regulation of defense response,” and “cellular response to salicylic acid stimulus.” Biotic stress terms—including “response to fungus,” “response to bacterium,” and “response to oomycetes”—were also significantly enriched at 4 weeks but were weak or absent at 3 weeks. These results indicate that 4-week-old *exo70b1-1* exhibits a distinct transcriptional profile enriched for SA/SAR- and broader immune-related pathways. This transcriptional pattern is consistent with the previously reported immune-associated background of *exo70b1-1* and suggests that its altered response to dark treatment may involve broader defense/stress signaling in addition to senescence-associated processes.

## 3. Discussion

Leaf senescence is a highly coordinated developmental program that enables nutrient reallocation through the integration of endogenous age cues and environmental signals [2,47,48]. However, the contribution of exocyst-mediated trafficking to this process remains poorly understood. In this study, we provide genetic, physiological, and transcriptomic evidence that *EXO70B1* contributes to leaf responses during DIS in an age-dependent manner in *Arabidopsis thaliana*. Loss of *EXO70B1* accelerated dark-induced leaf yellowing, chlorophyll degradation, photosynthetic decline, and tissue deterioration, with these effects being most pronounced in mature leaves (Figure 2). Genetic analysis further showed that the accelerated chlorophyll-loss phenotype of *exo70b1-1* under darkness was largely dependent on *NYE1*, a Mg-dechelatase required for chlorophyll catabolism, thereby placing *NYE1* genetically downstream of this response (Figure 5). In parallel, transcriptomic profiling revealed that mature *exo70b1-1* leaves underwent substantial defense-associated transcriptional reprogramming prior to visible damage, including activation of SA- and SAR-related pathways (Figure 6 and Figure 7). Together, these results support an age-dependent role for *EXO70B1* in maintaining leaf integrity during dark treatment and provide a framework for interpreting the enhanced sensitivity of mature *exo70b1* leaves to prolonged darkness.

### 3.1. EXO70B1 Is Associated with the Early Response to DIS

Our data reveal that *EXO70B1* expression increases as leaves mature (Figure 1A) and is transiently induced during the early phase of dark treatment (Figure 2A). The peak of *EXO70B1* transcripts at 6 h after dark transfer suggests that it may participate in the early response to sudden carbon limitation or darkness-associated stress (Figure 2A). This transient induction is consistent with a role for EXO70B1-mediated membrane trafficking in the initial adaptation to dark treatment. However, expression dynamics alone do not establish a direct regulatory role, and the upstream signals and downstream processes associated with this early induction remain to be defined. The subsequent decline in its expression during prolonged darkness coincides with the progression of senescence. Under these conditions, *exo70b1* mutants exhibited more pronounced dark-induced tissue deterioration than Col-0 at morphological, physiological, and molecular levels, including faster photosynthetic decline, greater membrane leakage, and elevated oxidative stress (Figure 2D–G). Taken together, these observations support the view that *EXO70B1* is associated with maintaining leaf integrity during dark treatment. Notably, this effect was strongly age dependent, being evident in mature plants but not in younger plants, suggesting that the contribution of *EXO70B1* to maintaining leaf integrity during dark treatment becomes more apparent in mature leaves.

### 3.2. NYE1 Is Required for Accelerated Chlorophyll Degradation in exo70b1 During Dark Treatment

To test whether the accelerated chlorophyll loss in *exo70b1* depends on the canonical *NYE1*-associated chlorophyll degradation pathway, we analyzed the *exo70b1-1 nye1-1* double mutant. Under dark treatment, the introduction of the *nye1-1* mutation largely suppressed the accelerated yellowing phenotype of *exo70b1-1* (Figure 5A). Moreover, chlorophyll content did not differ significantly between *nye1-1* and *exo70b1-1 nye1-1* under the tested conditions (Figure 5B). Together, these data indicate that the accelerated chlorophyll degradation observed in the *exo70b1-1* background during DIS is largely dependent on *NYE1*.

At the same time, these results do not support a direct regulatory connection between *EXO70B1* and the chlorophyll catabolic machinery itself. Rather, they place *NYE1* genetically downstream of the accelerated chlorophyll loss phenotype observed in *exo70b1-1* during dark treatment. In addition, the *nye1-1* mutation did not suppress the spontaneous necrotic lesion phenotype of *exo70b1* under standard growth conditions (Figure S4). Taken together, these observations indicate that *NYE1*-dependent chlorophyll degradation during DIS and the autoimmune-like lesion phenotype of *exo70b1* are at least partially genetically separable. Thus, although *NYE1* is required for the accelerated chlorophyll loss phenotype in the *exo70b1-1* background, it does not account for all defects associated with loss of *EXO70B1*.

### 3.3. Age-Dependent Transcriptomic Divergence Provides a Molecular Framework for Stage-Specific Sensitivity

Our stage-resolved RNA-seq analysis provides a molecular framework for the age-dependent hypersensitivity to DIS. The transcriptomic profiling reveals that while the *exo70b1* mutant is transcriptionally similar to Col-0 at 3 weeks, it undergoes substantial transcriptional reprogramming by week 4. This transition was characterized by the significant upregulation of SA signaling, SAR, and broader defense networks (Figure 6C, Figure 7B). These data indicate that mature *exo70b1* leaves enter a distinct defense- and stress-associated transcriptional state before visible tissue collapse.

Previous studies have linked *EXO70B1* to autophagy-related trafficking [24,25], and seedling-based assays under carbon-limiting conditions have not revealed an obvious hypersensitive phenotype in *exo70b1* (Figure 3A). Our experimental system differs from those assays, as mature plants were exposed to prolonged darkness rather than to a defined carbon-starvation treatment. Prolonged darkness is expected to reduce photosynthetic carbon input, but it also imposes a broader physiological challenge involving senescence progression, defense activation, metabolic adjustment, and altered cellular homeostasis. Because the present study did not directly quantify carbohydrate status or autophagic flux, the relative contributions of darkness-associated metabolic limitation and trafficking-related defects cannot be resolved here. Nevertheless, the transcriptomic state observed in mature *exo70b1* leaves prior to visible damage more directly supports a model in which these leaves are physiologically sensitized before dark treatment causes overt deterioration. In particular, premature activation of defense-associated programs may reduce the ability of mature leaves to maintain cellular homeostasis during prolonged darkness.

### 3.4. A Working Model for EXO70B1 Function in Mature Leaves During Prolonged Darkness

Taken together, our genetic, physiological, and transcriptomic analyses support a working model in which *EXO70B1* contributes to the maintenance of cellular homeostasis in mature leaves during prolonged darkness. In wild-type plants, *EXO70B1* may facilitate leaves accommodate the physiological demands associated with DIS, thereby preserving tissue integrity during dark treatment. In the *exo70b1* background, by contrast, mature leaves enter a defense-associated sensitized state prior to visible damage, particularly involving activation of SA- and SAR-related pathways. This preconditioned state may reduce the capacity of mature leaves to tolerate prolonged darkness, thereby accelerating chlorophyll loss, oxidative damage, membrane leakage, and tissue deterioration.

Within this framework, *NYE1* is genetically required for the accelerated chlorophyll-loss phenotype of *exo70b1*, but the present data do not support *EXO70B1* as a direct regulator of the chlorophyll catabolic machinery. Instead, loss of *EXO70B1* appears to create a mature-leaf context in which canonical chlorophyll degradation proceeds more rapidly under dark treatment. Previous studies showed that *EXO70B1* is linked to immune-related signaling, including TN2-dependent surveillance under standard growth conditions [34,36,38]. Although the involvement of this pathway in DIS was not tested here, these prior findings provide a plausible context for the defense-associated transcriptional state observed in mature *exo70b1* leaves.

At the same time, several mechanistic questions remain unresolved. The present study does not establish how *EXO70B1* deficiency promotes *NYE1*-dependent chlorophyll degradation, nor does it determine whether the defense-associated transcriptional state observed in mature *exo70b1* leaves is a primary driver of the phenotype or a secondary consequence of altered cellular homeostasis. In addition, although prolonged darkness is expected to reduce photosynthetic carbon input, the present experiments were not designed as a defined carbon-starvation assay, and carbohydrate status was not directly measured. Likewise, autophagic activity and flux were not assessed directly, leaving the contribution of altered autophagy-related recycling unresolved. Furthermore, because the RNA-seq analysis was performed at the bulk-tissue level, it cannot distinguish whether the defense-associated state arises uniformly across the leaf or preferentially within specific cell populations or physiologically compromised sectors. Accordingly, the current model should be regarded as a working framework rather than a definitive mechanistic explanation. This provides a basis for future work aimed at distinguishing the relative contributions of defense signaling, intracellular trafficking, and metabolic adjustment to age-dependent sensitivity to DIS during leaf senescence.

## 4. Materials and Methods

### 4.1. Plant Materials and Growth Conditions

All experiments used *Arabidopsis thaliana* ecotype Columbia-0 (Col-0) unless otherwise specified. The T-DNA insertion lines *exo70b1-1* (GK-114C03) and *exo70b1-2* (GK-156G02) were obtained from the GABI-Kat collection and genotyped as previously described [24]. Col-0 seeds were maintained in-house. The *nye1-1* mutant was obtained and verified as described [42]. To establish genetic relationships, we crossed *exo70b1-1* with *nye1-1* and identified homozygous double mutants by PCR genotyping and phenotypic analysis. Primers used for genotyping are listed in Supplementary Table S1.

Seeds were surface-sterilized in 75% (*v*/*v*) ethanol for 4 min followed by 100% ethanol for 1 min, dried on sterile filter paper, and sown on ½ MS medium plates (containing 1% *w*/*v* sucrose and 0.8% *w*/*v* agar, pH 5.8). After 48 h stratification at 4 °C in the dark, plates were placed vertically in growth chambers (22 °C, 10 h light/14 h dark, 150 µmol m^−2^ s^−1^) for 5 days. Seedlings were then transplanted to soil mixture comprising peat moss (PINDSTRUP Seeding Gold, 0–10 mm, pH 5.5, Latvia) and vermiculite at a 3:1 (*v*/*v*) ratio. Plants were maintained under short days (8 h light/16 h dark, 22 °C, 150 µmol m^−2^ s^−1^) and bottom-watered whenever the surface soil became slightly dry.

### 4.2. Plasmid Construction and Plant Transformation

To perform the genetic complementation assay, the *pEXO70B1::EXO70B1*-GFP construct was generated. A genomic fragment containing the 419-bp native promoter of *EXO70B1* and its coding sequence without the stop codon was amplified by PCR from Col-0 genomic DNA. The amplified fragment, flanked by EcoRI and SalI restriction sites, was first cloned into the pEASY-T1 Cloning Vector (TransGen Biotech Co., Ltd., Beijing, China). A GFP fragment carrying SalI and HindIII restriction sites was subsequently subcloned into the binary vector pCAMBIA1300 to generate pCAMBIA1300-GFP. The *pEXO70B1::EXO70B1* fragment was then excised using EcoRI and SalI and ligated upstream of GFP in pCAMBIA1300-GFP, yielding the final construct *pEXO70B1::EXO70B1*-GFP (hereafter referred to as *B1::B1-GFP*).

The construct was introduced into *Agrobacterium tumefaciens* strain EHA105 and transformed into *exo70b1-1* mutant plants using the floral dip method. Transgenic plants were selected on ½ MS medium containing hygromycin, and successful complementation was confirmed by phenotypic observation and RT-qPCR.

### 4.3. Treatment Conditions

To evaluate dark-induced responses under different developmental contexts, three distinct treatment assays were employed. For carbon starvation: seedlings initially grown on sucrose-containing ½ MS medium were transferred to sucrose-free ½ MS solid agar plates and maintained in continuous darkness. For the treatment of whole-plant: soil-grown plants were placed in black boxes externally shielded with black plastic bags to ensure absolute darkness. The lids of the boxes were loosely fitted to prevent complete hermetic sealing, and the boxes were kept in the same growth chamber as the control plants at 22 °C to maintain identical temperature and humidity. For the treatment of the detached-leaves: excised rosette leaves were placed on wet filter paper moistened with sterile deionized water in Petri dishes and incubated in complete darkness. The specific developmental stages of the plants used for each assay, the varying durations of dark treatment, and the exact sampling time points for subsequent physiological and molecular analyses are detailed in the respective figure legends.

### 4.4. Chlorophyll Quantification

Chlorophyll was extracted from pooled leaf tissue (comprising ≥ 3 plants per biological replicate) using a freshly prepared, light-protected solution of 95% acetone and absolute ethanol (2:1, *v*/*v*). Fresh leaves (W, g) were ground into a fine powder in liquid nitrogen and transferred to a centrifuge tube. An appropriate volume of extraction solution (V, mL) was added, and samples were mixed thoroughly by vortexing. After incubation at room temperature in the dark for 30–60 min (with tubes inverted 2–3 times during incubation), samples were centrifuged at 4 °C and 12,000 rpm for 2 min. Absorbance of the supernatant was measured at 645 nm and 663 nm using a UV-8000 spectrophotometer (Shanghai Metash Instruments Co., Ltd., Shanghai, China.) against a solvent blank. Total chlorophyll content was calculated using the following formula: Chlorophyll content (mg g^−1^ FW) = [(20 × OD_645_ + 8.02 × OD_663_) × V]/(1000 × W).

### 4.5. Photochemical Efficiency of PSII (Fv/Fm)

The maximum quantum efficiency of PSII (*Fv*/*Fm*) was measured on intact, attached 5th and 6th rosette leaves using a Handy PEA chlorophyll fluorometer (Hansatech Instruments, UK; sourced via HANSHATECH Scientific Instruments Co., Ltd., Tai’an, China). Leaves were dark-adapted for 15 min prior to measurement. A saturating light pulse (3500 µmol m^−2^ s^−1^) with a duration of 2 s was applied to determine the maximal fluorescence (Fm). For each treatment group, valid measurements were obtained from at least 5 independent leaves to serve as biological replicates.

### 4.6. RNA Extraction and RT-qPCR

Total RNA was extracted with RNAprep Pure Kit (Tiangen, Beijing, China), reverse-transcribed with Evo M-MLV (Accurate Biology), and analyzed on a Bio-Rad CFX Duet system using SYBR Green Pro Taq HS premix in a 20 µL reaction volume. The PCR cycling conditions consisted of an initial denaturation at 95 °C for 5 min, followed by 40 cycles of 95 °C for 30 s and 56 °C for 45 s. A melt curve analysis (56 °C to 95 °C, with a 0.5 °C increment every 5 s) was subsequently performed to verify primer specificity. ACTIN2 was used as the internal reference gene. Relative transcript abundance for each sample was calculated using the 2^−ΔCt^ method, except for the genetic complementation analysis (Figure 4A), which was calculated using the 2^−ΔΔCt^ method normalized to Col-0. Data represent the mean ± SD from three independent biological replicates. Primers used for RT-qPCR are listed in Supplementary Table S1.

### 4.7. Ion Leakage Assay

Leaf segments (0.1–0.2 g) were vacuum-infiltrated in deionized water, and shaken gently for 3 h at 25 °C, after which the initial conductivity (EC_1_) was recorded. Samples were then boiled for 15 min and cooled to room temperature before measuring the final conductivity (EC_2_). A water-only blank provided EC_0_. Relative conductivity = (EC_1_ − EC_0_)/(EC_2_ − EC_0_) × 100%. Ion permeability (%) = (Treated relative conductivity-Control relative conductivity)/(1 − Control relative conductivity) × 100%. Each treatment had ≥3 replicates.

### 4.8. DAB Staining

To detect H_2_O_2_ accumulation, a 3,3′-Diaminobenzidine (DAB) working solution (1 mg mL^−1^) was prepared by dissolving DAB powder in PBS buffer (pH 7.4) at 60 °C. Intact 5th and 6th rosette leaves from plants subjected to 3 days of whole-plant dark treatment were completely immersed in the DAB solution and vacuum-infiltrated for 30 s per cycle. This process was repeated 2–3 times until the leaves completely sank to the bottom. Samples were then incubated in the dark at room temperature or 28 °C for 3 h until brown spots became clearly visible. Leaves were rinsed with deionized water 2–3 times, and then transferred to 95% ethanol. Samples were heated in an 80–95 °C water bath until the leaves are completely destained (chlorophyll removed) and the spots are clearly visible. Destained leaves were placed on a white background for observation and photography.

### 4.9. RNA Sequencing and Transcriptomic Analysis

Total RNA was extracted from the 5th and 6th rosette leaves of 3-week-old and 4-week-old Col-0 and *exo70b1-1* plants using the RNAprep Pure Plant Plus Kit (Polysaccharides & Polyphenolics-rich, DP441, TIANGEN, Beijing, China), with three biological replicates per group. Poly(A) mRNA enrichment and fragmentation were performed using VAHTS mRNA Capture Beads (N401-01/02, Vazyme, Nanjing, China), substituting the corresponding step in the Fast RNA-seq Lib Prep Kit V2 (RK20306, ABclonal, Wuhan, China). The subsequent steps of RNA-seq library construction were completed using the ABclonal kit according to the manufacturer’s protocol. The resulting libraries were sequenced on a DNBSEQ-T7 sequencer (MGI, Shenzhen, China) to generate 150-bp paired-end reads.

The nf-core framework [49] was employed for the standardization of RNA-seq bioinformatic analyses. Specifically, the nf-core/rnaseq pipeline (v3.22.2) [49] was utilized for raw data quality control (assessed via FastQC and MultiQC), alignment to the *Arabidopsis thaliana* reference genome (Ensembl Plants release 62), and gene quantification. Differential expression analysis was subsequently performed using the nf-core/differentialabundance pipeline (v1.0.0) [49]. Differentially expressed genes (DEGs) were identified strictly based on the thresholds of adjusted *p* value (padj) ≤ 0.01 and |log_2_FoldChange| ≥ 1.5. Functional enrichment analysis of the DEGs was performed using Metascape (v3.5.20260201) [50]. Comprehensive mapping statistics and the complete list of DEGs are summarized in Supplementary Table S2.

### 4.10. Statistical Analysis

Unless otherwise indicated, all experiments were performed with at least three biological replicates. Data are presented as mean ± SD. Statistical analyses were performed using OriginPro 2024 SR1 10.1.0.178 (OriginLab, Northampton, MA, USA). Differences among multiple groups were analyzed by one-way analysis of variance (ANOVA) followed by Tukey’s multiple-comparison test. Significant differences were considered at *p* < 0.05. Grouping letters and pairwise comparison labels shown in the figures were generated using the Paired Comparison Plot app in Origin.

## Data Availability

The original contributions presented in this study are included in the article/Supplementary Materials. Further inquiries can be directed to the corresponding author.

## Supplementary Materials

The following supporting information can be downloaded at: https://www.mdpi.com/article/10.3390/plants15101461/s1, Figure S1: Spatiotemporal expression profile of the *Arabidopsis thaliana ATEXO70B1* (AT5G58430/*EXO70B1*) gene visualized by eFP Browser. Figure S2: *EXO70B1* negatively regulates dark-induced leaf senescence; Figure S3: Phenotypes of Col-0, *exo70b1-1*, and the complementation line *B1::B1-GFP* under natural conditions; Figure S4: Partial suppression of spontaneous lesion formation in the *exo70b1-1 nye1-1* double mutant. Table S1: List of primers used in this study: Table S2: DESeq2. results.
